# Biological and environmental contributions to the growth rates of preterm children aged 0–2 years: a birth cohort study

**DOI:** 10.3389/fpubh.2026.1544227

**Published:** 2026-04-29

**Authors:** Jiangshan Cao, Juan Li, Yan Li, Zhonggui Xiong

**Affiliations:** 1 Maternal and Child Health Hospital of Hubei Province, Tongji Medical College, Huazhong University of Science and Technology, Wuhan, China; 2 Medical College, Wuhan University of Science and Technology, Wuhan, China

**Keywords:** biological and environmental factors, growth rates, IGF-1 and IGFBP-3, parameter model, preterm children

## Abstract

**Objective:**

This study aimed to clarify biological and environmental factors contributing to the growth rates of preterm children.

**Methods:**

Multivariate linear regression analysis was used to identify the influence of biological and environmental factors on the reference values for the growth rates and the expression levels of serum IGF-1 and IGFBP-3 in preterm children.

**Results:**

This study revealed that the growth rates of length and weight in preterm children were significantly lower than those in full-term children during the first 6 months of age, and the growth rate of head circumference in preterm children was significantly lower than that in full-term children during the first 3 months of age (*p* < 0.05), and gradually reached the levels of full-term children over time (*p* > 0.05). Similarly, the expression levels of serum IGF-1 and IGFBP-3 in preterm children were significantly lower than those in full-term children during the first 12 months of age (*p* < 0.05), and gradually reached the levels of full-term children over time (*p* > 0.05). In addition, this study found that biological and environmental factors had a significant impact on the growth rates of preterm children.

**Conclusion:**

This study established scientific parameter models for promoting growth and development of preterm children.

## Introduction

The longitudinal follow-up of the growth rates involves regularly physical measurements to screen preterm children who deviate from the normal trajectory of growth and development. As for preterm children, there are two ways of the growth rate, such as complete and partial catch-up growth. Some preterm children show complete catch-up growth, and their growth and development reach to the level of normal trajectory, while others show partial catch-up growth, and their growth and development still deviate from the level of normal trajectory. So far, optimal growth rates are critical for future growth and development of preterm children (1).

The growth rates of preterm children are nonlinear and varied, with alternating periods of rapid growth and stagnation. Previous studies have identified the growth rates of preterm children as a crucial area for basic research in the field of growth and development (2). In 2018, the INTERGROWTH-21st Project collected only a large sample of cross-sectional data to establish international references for child growth and development (3). At present, few studies used longitudinal follow-up to establish national or regional references of child growth rates, especially for preterm children (4). Therefore, the growth rates of preterm children are a valuable basic research of growth and development, compared with cross-sectional investigation.

However, it is important to note that the growth patterns of preterm children differ significantly from those of full-term children at different ages. Previous research has also shown that the growth rates of preterm children are regulated by the expression levels of serum IGF-I and IGFBP-3 through the GH/IGF-1 axis (5). However, the regulatory mechanism of the growth rates in preterm children is not still clear. GH adjusts the growth rates of preterm children in a pulsating pattern, whereas IGF-1 only forms the binary complex with IGFBP-3 to release free IGF-1 in the target organs of growth and development (6). Hence, the expression levels of serum IGF-I and IGFBP-3 are closely correlated with the growth rates of preterm children.

Importantly, both biological and environmental factors play a significant role in the growth rates of preterm children. Perinatal factors were the important biological and environmental factors for the growth rates of preterm children, such as pregnancy times, gestational age, delivery times, delivery pattern, and hyperbilirubinemia (7). Specific pituitary structures and/or functions are damaged because of the interaction of biological and environmental factors, thereby regulating the expression levels of serum IGF-1 and IGFBP-3, and in turn adjusting the growth rates of preterm children (8).

**Figure 1:**
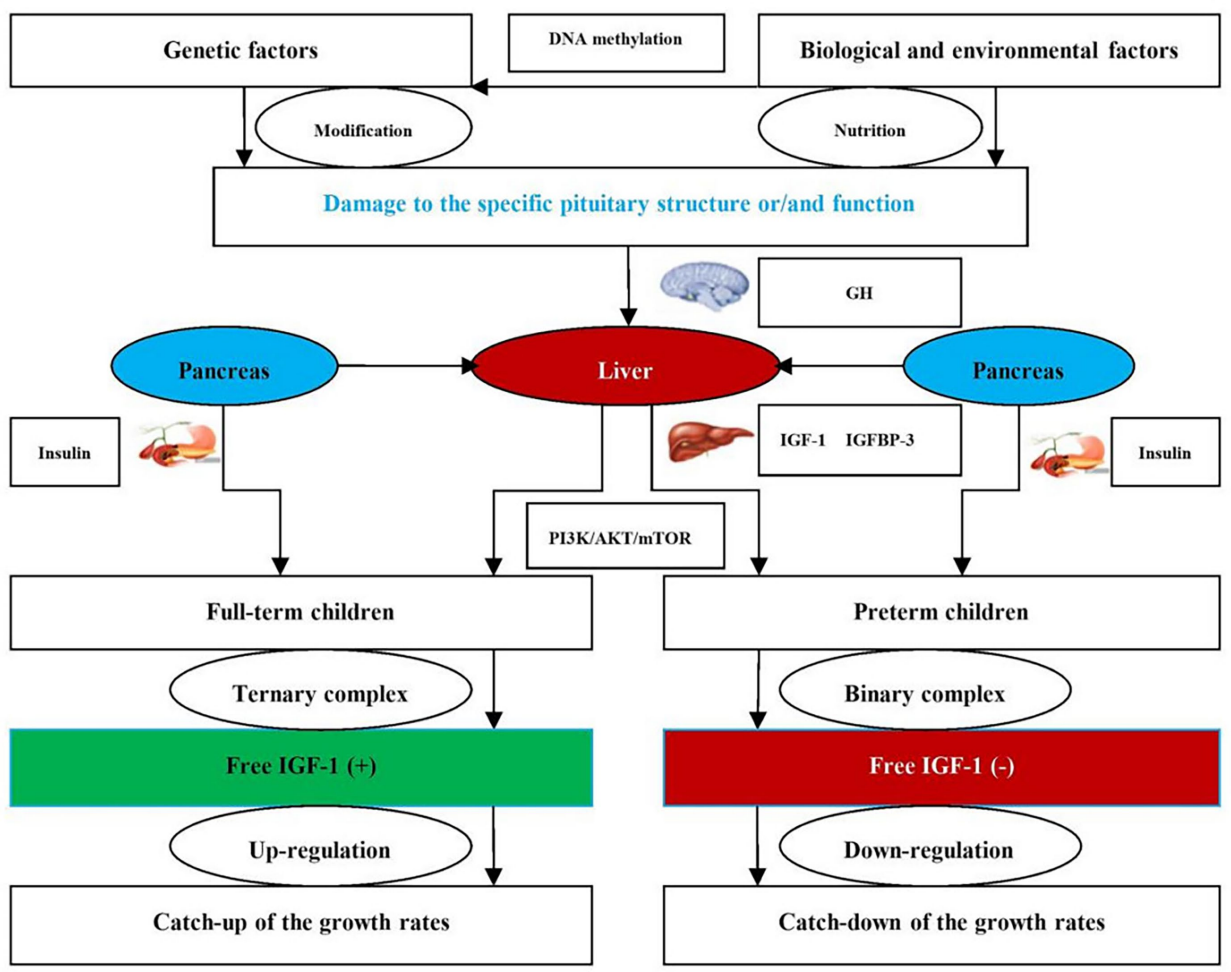
Flow chart of regulatory mechanism on the growth rates between preterm and full-term children. GH, growth hormone; IGF-1, insulin-like growth factor-1; IGFBP-3, insulin-like growth factor binding protein-3; PI3K/AKT/mTOR, phosphoinositide-3-kinase/protein kinase B/mammalian target of rapamycin; signaling pathway related to the growth rates between preterm and full-term children; ternary complex: composed of IGF-1, BPs, and ALS; binary complex: composed of IGF-1 and BPs; +, expressed as increase in free IGF-1; −, expressed as decrease in free IGF-1.

To address this gap, we collected longitudinal data from a specific birth cohort of preterm and full-term children during the first 2 years of life. Our study aimed to clarify the damage to specific pituitary structures and/or functions caused by the interaction between biological and environmental factors, thereby regulating the expression levels of serum IGF-1 and IGFBP-3, and in turn adjusting the growth rates of preterm children (Figure 1). On this basis, scientific intervention measures should be formulated to promote future growth and development of preterm children.

## Materials and methods

### Study subjects

In this study, 339 paired preterm and full-term children (183 boys and 156 girls) were enrolled at the Maternal and Child Health Hospital of Hubei Province and Jingzhou City between January 2021 and December 2023. All participants were Han Chinese living in the urban areas of Hubei Province. They were matched by gender, age (within ± 7 days), and residential community. All data were collected during routine health visits of preterm and full-term children.

According to the research design, the inclusion criteria were singleton live births of preterm children with gestational ages between 28 + 0 and 36 + 6 weeks in the case group, and full-term children with gestational ages between 37 + 0 and 42 + 6 weeks in the control group. The exclusion criteria were as follows: (1) twins or multiples; (2) severe congenital malformation (a type of morphological defect with abnormal development of internal organs) or genetic metabolic disease (a type of genetic disease with congenital deficiency of metabolic functions) at birth; (3) non-Chinese parents; (4) maternal height < 145 cm; (5) maternal age < 18 years, or > 40 years; (6) mothers who had taken adrenocortical hormones or immunosuppressants continuously for more than 1 month during pregnancy; and (7) mothers who had suffered from severe anemia, diabetes, preeclampsia, eclampsia, hyperthyroidism, hypothyroidism, or chronic hypertension during pregnancy (9).

### Clinical measurements

The length, weight, and head circumference of preterm and full-term children were regularly monitored at birth and at 1, 3, 6, 9, 12, 15, 18, 21, and 24 months of age using standard physical measurement methods. This follow-up period consisted of 10 monitoring time points: the first time point was within 24 h after birth, as determined by the provincial and municipal delivery institutions (administrated by the provincial and municipal health committee, respectively), and the remaining time points were at specific months of physical measurements, as determined by the maternal and child health institutions. The length and weight of preterm and full-term children were measured in a supine position using a height/weight appliance (Kangwa, Wuhan, China; length range 30–105 cm with digit counter readings precise to 0.1 cm, and weight range 0–60 kg with digit counter readings precise to 0.01 kg). The head circumference of preterm and full-term children was measured in a seated position using a soft tape (Kangwa, Wuhan, China; head circumference range 20–55 cm with digit counter readings precise to 0.1 cm). Finally, the average values of the two physical measurements were calculated at each time point.

### Experimental research

The serum IGF-1 and IGFBP-3 of preterm and full-term children were measured at birth and at 6, 12, 18, and 24 months of age using ELISA. This follow-up period consisted of five monitoring time points chosen to coincide with the key months for physical measurements, as determined by the provincial and municipal maternal and child health institutions. Peripheral anticoagulant venous blood (3 mL) was collected from all subjects before 10 a.m. every day, and all samples were stored in a − 80 °C freezer for about 1 month. The expression levels of serum IGF-1 and IGFBP-3 were tested regularly using reagent kits (DSL abs, United States). Finally, the expression levels of serum IGF-1 and IGFBP-3 were measured by the absorbance of optical density (OD) at a wave length of 450 nm.

### Epidemiological investigation

The biological and environmental factors of preterm and full-term children were investigated at 24 months of age using self-designed survey questionnaire. These influencing factors were as follows: (1) general demographic information, such as child’s name, gender, and age; (2) family conditions, such as parental height, occupation, education level, family upbringing pattern, economic status, and other family conditions; (3) maternal characteristics during pregnancy, such as smoking, alcoholism, drug dependence, assisted reproduction, gestational age, delivery pattern, and other perinatal conditions; (4) child characteristics at birth, such as length, weight, head circumference, Apgar score, feeding pattern, supplementary food addition, and other nutritional status; and (5) any previous health history of child and family diseases (Table 1) (10, 11). Finally, these influencing factors were categorized as either positive variables (facilitating effect) or negative variables (inhibiting effect) on the growth rates of preterm children.

**Table 1:** Variable description between preterm and full-term children.

Variable	Description	Classification	Definition
Gender	Qualitative	Male	N/A
		Female	
Family population	Quantitative	Person	Total number of family members living together
Monthly income	Quantitative	Yuan	Monthly per capita income
Birth length	Quantitative	cm	Length at birth
Birth weight	Quantitative	kg	Weight at birth
Birth head circumference	Quantitative	cm	Head circumference at birth
Gestational age	Quantitative	Week	A lot of weeks between the first day of last menstrual period and current pregnancy
Pregnancy times	Quantitative	1	N/A
		2	
		3 and more	
Delivery times	Quantitative	1	N/A
		2	
		3 and more	
Delivery pattern	Qualitative	Vaginal delivery	N/A
		Cesarean delivery	
Birth asphyxia	Qualitative	Yes	A kind of disease in which a newborn infant is unable to breathe normally after birth
		No	
Hyperbilirubinemia	Quantitative	μmol/L	A kind of disease in which serum bilirubin exceeds the normal level of the newborn infant

### Quality control

Strict quality control measures were implemented in accordance with the established guidelines. The following specific methods were used for quality control measures: (1) measurement calibration was performed before conducting daily clinical observations; (2) each child was measured twice by two investigators using standard physical measurement methods. All the subjects and two investigators did not know the research grouping of preterm and full-term children (double-blind method). Discrepancies between the two measurements for length, weight, and head circumference were limited to 0.1 cm, 0.01 kg, and 0.1 cm, respectively. If the discrepancies exceeded the corresponding limits, a third measurement was taken by another investigator; (3) a preliminary review and re-examination were conducted after physical measurements; and (4) the missing rate was kept below 10%.

### Statistical analysis

Epidata 4.4 (Epidata Association, Odense, Denmark) was used to establish the related database, and SPSS 20.0 (IBM Corp., Armonk, New York, United States) was used to conduct the logic check and statistical analysis. The growth rates of preterm and full-term children were in accordance with the normal distribution of quantitative data.

In the descriptive analysis, the growth rates of length, weight, and head circumference are defined as follows: *Y* = (*X*_i_ – *X*_i-1_) / (*M*_i_ – *M*_i-1_). Here, *Y* represents the growth rates of length, weight, and head circumference, *X*_i_ is the absolute values of physical measurements in a specific age group, *X*_i-1_ is those in the adjacent previous age group, *M*_i_ is the absolute values of month age in the specific age group, and *M*_i-1_ is those in the adjacent previous age group. Differences in the growth rates between preterm and full-term children are expressed as mean and 95% CI. A 1:1 paired *t*-test was used to compare the growth rates between preterm and full-term children, with a significance level of *p* < 0.05.

Based on the descriptive analysis, parameter models for the growth rates of length, weight, and head circumference are defined as follows: *Y* = *β*_1_*X*_1_ + *β*_2_*X*_2_ + … + *β*_i_X_i_ + *C*, where *Y* represents the dependent variable (the growth rates of length, weight, and head circumference), *X*_i_ represents the independent variables (biological and environmental factors), *β*_i_ is the standardized coefficient, and *C* is a constant. Multivariate linear regression analysis was used to determine the impact of these influencing factors on the growth rates of length, weight, and head circumference in preterm children, with a significance level of *p* < 0.05.

## Results

### Baseline characteristics

Of 339 paired preterm and full-term children (183 boys and 156 girls), 23 participants were lost to follow-up (13 boys and 10 girls), resulting in a missing rate of 6.78% in the longitudinal follow-up study. Nevertheless, there was not a selective bias in the demographic characteristics between preterm and full-term children (Table 2).

**Table 2:** Demographic characteristics between preterm and full-term children.

Characteristics	Classification	Total number (n)	Missing number (n)	Missing rate (%)
Gender	Male	183	13	7.10
	Female	156	10	6.41
Region	Wuhan City	225	16	7.11
	Jingzhou City	114	7	6.14
Total		339	23	6.78

### Growth rates of length, weight, and head circumference

**Figure 2:**
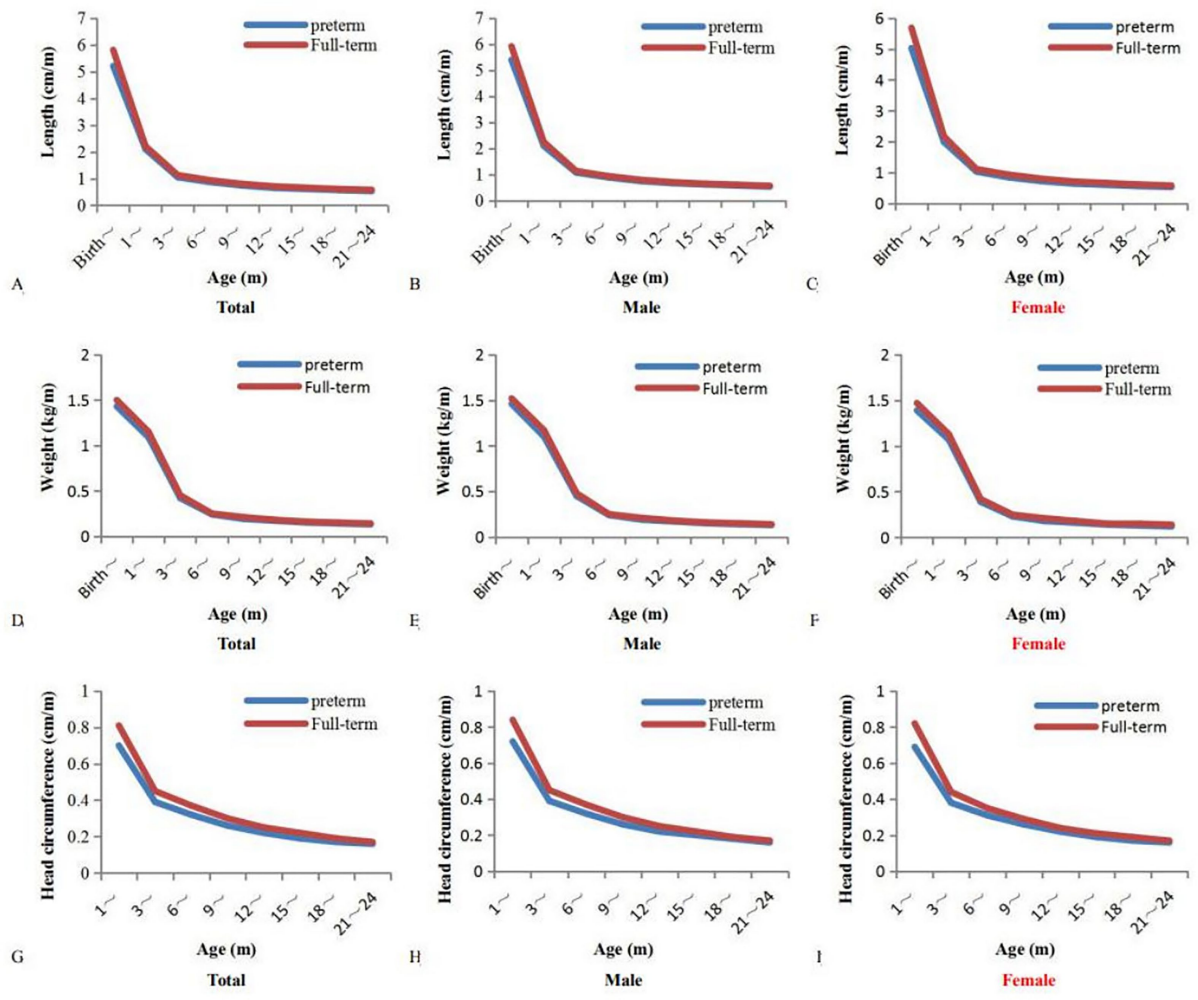
Growth rates of age-specific length (cm/m), weight (kg/m), and head circumference (cm/m) between preterm and full-term children. Growth rates: relative values for increase in length, weight, and head circumference per month. **(A-C)**: Growth rates of age-specific length (cm/m) between preterm and full-term children; **(D-F)**: Growth rates of age-specific weight (kg/m) between preterm and full-term children; **(G-I)**: Growth rates of age-specific head circumference (cm/m) between preterm and full-term children. The growth rates of length and weight within the first 6 months: *p* < 0.05, the growth rate of head circumference within the first 3 months: *p* < 0.05.

The growth rates of length and weight in preterm children were significantly lower than those in full-term children within the first 6 months of age (*p* < 0.05), and the growth rate of head circumference in preterm children was significantly lower than that in full-term children during the first 3 months of age (*p* < 0.05). With increasing age, there was no significant difference in the growth rates of length, weight, and head circumference between preterm and full-term children (*p* > 0.05) (Figure 2).

### Expression levels of IGF-1 and IGFBP-3

**Figure 3:**
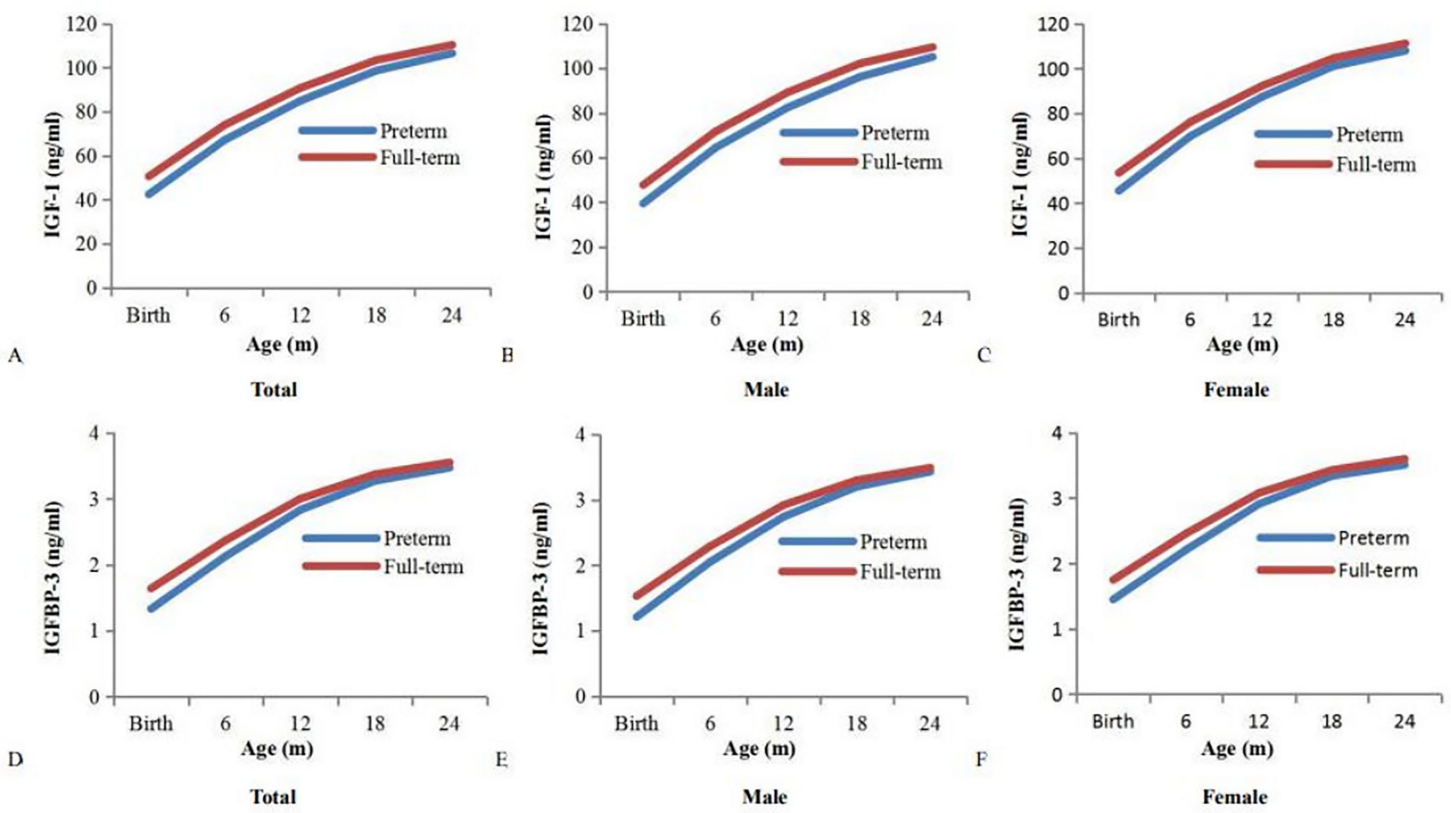
Expression levels of age-specific IGF-1 and IGFBP-3 (ng/ml) between preterm and full-term children. IGF-1: insulin-like growth factor-1; IGFBP-3: insulin-like growth factor binding protein-3; expression levels: serum concentration of IGF-1and IGFBP-3 at birth and at 6, 12, 18, and 24 months of age. **(A-C)**: Expression levels of age-specific serum IGF-1 (ng/ml) between preterm and full-term children; **(D-F)**: Expression levels of age-specific serum IGFBP-3 (ng/ml) between preterm and full-term children. The expression levels of serum IGF-1 and IGFBP-3 within the first 12 months of age: *p* < 0.05.

The expression levels of serum IGF-1 and IGFBP-3 in preterm children were significantly lower than those in full-term children during the first 12 months of life (*p* < 0.05). With increasing age, there were no significant differences in the expression levels of serum IGF-1 and IGFBP-3 between preterm and full-term children (*p* > 0.05) (Figure 3).

### Biological and environmental factors of the growth rates

Taking Y (the growth rates of length, weight, and head circumference) as the dependent variable, and X_i_ (biological and environmental factors) as the independent variables, there were several positive variables (facilitating effect) and negative variables (inhibiting effect) that showed significant differences in their contributions to the growth rates of length, weight, and head circumference in preterm children (Tables 3–5).

**Table 3:** Influencing factors for the growth rate of preterm children by age-specific length (cm/m).

Model	Non-standardized coefficient		Standardized coefficient	*t*	*P*	Collinearity statistics	
	βn	SD	βs			δ	VIF
Constant	10.610	3.545		2.993	0.003		
Group	0.102	0.019	0.361	5.450	0.000	0.870	1.150
Birth length	0.167	0.012	0.636	13.898	0.000	0.356	2.807
Gestational age	0.980	0.070	0.769	13.938	0.000	0.352	2.842
Hyperbilirubinemia	−0.758	0.302	−0.122	2.510	0.013	0.823	1.890
Family population	−0.333	0.153	−0.132	2.168	0.031	0.732	1.578
Delivery pattern	−0.024	0.012	−0.154	2.153	0.037	0.389	1.211
Pregnancy times	−0.315	0.161	−0.108	2.103	0.043	0.334	1.022
Delivery times	−0.038	0.021	−0.149	2.012	0.047	0.433	1.013

Parameter model (length): *Y* = 0.167X_1_ + 0.980X_2_ − 0.758X3 − 0.333X_4_ − 0.024X_5_ − 0.315X_6_ − 0.038X_7_ + 10.610, *Y* represents the growth rate of length in preterm children, X_1_ to X_7_ represents birth length, gestational age, hyperbilirubinemia, family population, delivery pattern, pregnancy times, and delivery times, respectively.

βn, non-standardized coefficient; βs, standardized coefficient; SD, standard deviation; δ, tolerance error; VIF, variance inflation factor; +, expressed as the positive variables (facilitating effect); −, expressed as the negative variables (inhibiting effect).

**Table 4:** Influencing factors for the growth rate of preterm children by age-specific weight (kg/m).

Model	Non-standardized coefficient		Standardized coefficient	*t*	*P*	Collinearity statistics	
	βn	SD	βs			δ	VIF
Constant	4.711	0.715		6.590	0.000		
Group	0.181	0.025	0.383	5.852	0.000	0.885	1.170
Birth weight	0.161	0.022	0.536	11.324	0.000	0.356	2.807
Gestational age	0.203	0.014	0.773	14.343	0.000	0.885	1.130
Gender	−1.720	0.057	−715	3.023	0.003	0.983	1.017
Hyperbilirubinemia	−0.130	0.061	−0.102	2.141	0.033	0.987	1.013
Delivery times	−0.029	0.014	−0.180	2.105	0.037	0.490	1.124
Family population	−0.018	0.009	−0.208	2.056	0.041	0.334	1.036

Parameter model (weight): *Y* = 0.161X_1_ + 0.203X_2_ − 1.720X_3_ − 0.130X_4_ − 0.029X_5_ − 0.018X_6_ + 4.711, *Y* represents the growth rate of weight in preterm children, X_1_ to X_6_ represents gestational age, birth weight, gender, hyperbilirubinemia, delivery times, and family population, respectively.

βn, non-standardized coefficient; βs, standardized coefficient; SD, standard deviation; δ, tolerance error; VIF, variance inflation factor; +, expressed as the positive variables (facilitating effect); −, expressed as the negative variables (inhibiting effect).

**Table 5:** Influencing factors for the growth rate of preterm children by age-specific head circumference (cm/m).

Model	Non-standardized coefficient		Standardized coefficient	*t*	*P*	Collinearity statistics	
	βn	SD	βs			δ	VIF
Constant	3.233	0.022		20.212	0.000		
Group	−0.988	0.180	−0.353	5.497	0.000	0.857	1.270
Head circumference	0.213	0.018	0.752	9.880	0.000	0.376	2.107
Gender	−0.459	0.165	−0.188	2.777	0.006	0.652	1.247
Gestational age	0.120	0.045	0.201	2.643	0.009	0.739	1.354
Monthly income	0.348	0.138	0.172	2.523	0.012	0.812	1.367
Hyperbilirubinemia	−0.586	0.348	−0.122	2.241	0.023	0.987	1.013
Pregnancy times	−0.200	0.104	−0.146	2.121	0.046	0.744	1.345

Parameter model (head circumference): *Y* = 0.213X_1_ − 0.459X_2_ + 0.120X_3_ + 0.348X_4_ − 0.586X_5_ − 0.200X_6_ + 3.233, *Y* represents the growth rate of head circumference in preterm children, X_1_ to X_6_ represented head circumference, gender, gestational age, monthly income, hyperbilirubinemia, and pregnancy times, respectively.

βn, non-standardized coefficient; βs, standardized coefficient; SD, standard deviation; δ, tolerance error; VIF, variance inflation factor; +, expressed as the positive variables (facilitating effect); −, expressed as the negative variables (inhibiting effect).

## Discussion

In recent years, the growth rates of preterm children have significantly improved owing to improvement in healthy conditions and close collaboration between obstetrics and pediatrics (12). A study has identified a critical “growth window” for the growth rates of preterm children within the first 3 months of corrected age, which greatly impacts their future growth and development (13). However, another study has shown that the growth rates of preterm children differ significantly from those of full-term children because of the increased risk of cumulative malnutrition and fetal growth restriction (14). Compared with full-term children, catch-up growth of preterm children highlighted the first growth peak within the first 3 months of age.

The growth rates of preterm children are a crucial area of basic research in the field of growth and development (15). However, the growth rates of preterm children differ significantly from those of full-term children at different ages. A study has identified two distinct growth patterns in preterm children, such as complete and partial catch-up growth. Some preterm children present complete catch-up growth, and their growth and development reach to the level of normal trajectory, while others present partial catch-up growth, and their growth and development still deviate from the level of normal trajectory. Therefore, the goal for preterm children is to approach the growth rates of full-term children within the first year of life in the light of growth patterns (16, 17).

The growth trajectories of preterm children differ significantly from those of full-term children within the first 6 months of life (18). This study found that the growth rates of preterm children were significantly lower than those of full-term children. Previous research has shown that catch-up growth of preterm children is often hindered by cumulative malnutrition and fetal growth restriction compared with full-term children (19, 20). One plausible hypothesis is that preterm children have a higher probability of admission to the Neonatal Intensive Care Unit (NICU) because of preterm birth, low birth weight, and fetal growth restriction (21). Another plausible hypothesis is that preterm children have a poor function of placental nutrient transfer due to chorionicity, placental fusion, and central and peripheral insertion of umbilical cord. On these hypotheses, nutritional and mineral deficiencies inevitably affect the growth rates of preterm children. Additional calories and minerals may regulate heat metabolism through the OXPHOS signaling pathway (22). Supplementation of preterm formula or breast milk fortifiers may be beneficial for preterm children after being discharge from hospital (23–25).

However, the growth trajectories of preterm children are similar to those of full-term children after 6 months of age (26). This study also found that the growth rates of preterm children gradually reached those of full-term children. Arora et al. (27) reported there were not significant differences in the growth rates between preterm and full-term children after 6 months of age due to early intervention measures. Catch-up growth is attributed to the importance of continuous breastfeeding and the timely introduction of solid foods to meet the heat requirements of preterm children (27). The proportion of protein, fat and lactose in breast milk is adjusted with the growth stage of preterm infants. For example, colostrum (2–3 days of postpartum) is rich in antibodies and immune factors, transitional milk (4–10 days of postpartum) is rich in fat, and mature milk (after 11 days of postpartum) is rich in long-term nutritional supplementation. Importantly, it is critical for preterm children to continue breastfeeding and introduce solid foods in a timely manner to effectively promote growth and development (28).

The expression levels of IGF-I and IGFBP-3 have been pivotal in the growth rates of preterm children (29). This study showed that the expression levels of serum IGF-1 and IGFBP-3 in preterm children were significantly lower than those in full-term children aged from birth to 12 months, and gradually reached the levels of full-term children over time. Previous research has also shown that the growth rates of preterm children are affected by the expression levels of serum IGF-I and IGFBP-3 (30). Specifically, the growth rates of preterm children are regulated by the GH/IGF-1 axis through the PI3K/AKT/mTOR signaling pathway (31, 32). The regulatory mechanism is that GH adjusts the growth rates of preterm children in a pulsating pattern, whereas IGF-1 forms the ternary or binary complex with IGFBP-3 and ALS to release free IGF-1 in the target organs of growth and development (33). The free IGF-1 of full-term children is up-regulated due to the ternary complex formed by IGF-1, IGFBP-3, and ALS. However, the free IGF-1 of preterm children is down-regulated due to the binary complex formed by IGF-1 and IGFBP-3. Therefore, the expression levels of serum IGF-I and IGFBP-3 are closely correlated with the growth rates of preterm children (34).

Furthermore, biological and environmental factors play a significant role in the growth rates of preterm children (35, 36). This study also identified several positive and negative variables that had a significant impact on the growth rates of preterm children. Specifically, perinatal factors were the key biological and environmental factors for the growth rates of preterm children, such as pregnancy times, gestational age, delivery times, delivery pattern, and hyperbilirubinemia. Additionally, nutritional exposures were also the positive determinants for the growth rates of preterm children, such as birth length, weight, and head circumference. Zhang et al. (37) clarified the interaction of biological and environmental factors on the growth rates of preterm children. In addition to gene polymorphisms, specific pituitary structures and/or functions are damaged by biological and environmental factors through DNA methylation, thereby regulating the expression levels of serum IGF-1 and IGFBP-3, and in turn adjusting the growth rates of length, weight, and head circumference through the PI3K/AKT/mTOR signaling pathway (38–41). Based on biological and environmental factors, scientific intervention measures should be formulated to improve the growth rates of preterm children. For example, it is necessary for close collaboration between obstetrics and pediatrics, technological innovation of NICU, and nutritional supplementation of preterm formula or breast milk fortifiers to promote the growth rates of preterm children.

This study had some limitations. One major limitation was that we were unable to monitor all participants, which prevented us from obtaining more comprehensive information on the growth rates of preterm children aged from birth to 3 years. This was mainly due to the loss of health visits at the specific time points for physical measurements.

In future studies, a birth cohort study should be conducted to monitor the growth rates of preterm children over a prolonged period of longitudinal follow-up. Furthermore, a multi-center study should be conducted to further clarify the growth rates of preterm children with a larger sample size and stratified analysis. Therefore, longitudinal follow-up of the growth rates is a valuable basic research for growth and development of preterm children, compared with cross-sectional investigation.

## Conclusion

In general, the growth rates of preterm children are a crucial area of basic research in the field of growth and development. This study clarified the parameter models for biological and environmental factors in relation to the reference values for the growth rates, as well as the expression levels of IGF-1 and IGFBP-3 in preterm children using multivariate linear regression analysis. Importantly, specific pituitary structures and/or functions are damaged by biological and environmental factors through DNA methylation, thereby regulating the expression levels of serum IGF-1 and IGFBP-3, and in turn adjusting the growth rates of length, weight, and head circumference through the PI3K/AKT/mTOR signaling pathway. Therefore, it should be necessary to provide scientific intervention measures for the growth rates of preterm children.

## Data Availability

The original contributions presented in the study are included in the article/supplementary material, further inquiries can be directed to the corresponding author.

## References

[1] Vinther JL Ekstrøm CT Sørensen TI Cederkvist L Lawlor DA Andersen AN. Gestational age and trajectories of body mass index and height from birth through adolescence in the Danish National Birth Cohort. Sci Rep. (2023) 13:3298. doi: 10.1038/s41598-023-30123-y, 36843043 PMC9968714

[2] Han J Jiang Y Huang J Zhang Y Zhang Y Zhang Y . Postnatal growth of preterm infants during the first two years of life: catch-up growth accompanied by risk of overweight. Ital J Pediatr. (2021) 47:66. doi: 10.1186/s13052-021-01019-2, 33726805 PMC7968173

[3] Villar J Ismail LC Urias ES Giuliani F Ohuma EO Victora CG . The satisfactory growth and development at 2 years of age of the INTERGROWTH-21st fetal growth standards cohort support its appropriateness for constructing international standards. Am J Obstet Gynecol. (2018) 218:S841–54. doi: 10.1016/j.ajog.2017.11.564, 29273309 PMC5807090

[4] Giuliani F Cheikh Ismail L Bertino E Bhutta ZA Ohuma EO Rovelli I . Monitoring postnatal growth of preterm infants: present and future. Am J Clin Nutr. (2016) 103:635S–47S. doi: 10.3945/ajcn.114.106310, 26791186 PMC6443302

[5] Neves PAR Vaz JS Maia FS Baker P Gatica-Domínguez G Piwoz E. Rates and time trends in the consumption of breast milk, formula, and animal milk by children younger than 2 years from 2000 to 2019: analysis of 113 countries. Lancet Child dolesc Health. (2021) 5:619–30. doi: 10.1016/S2352-4642(21)00163-2, 34245677 PMC8376656

[6] Makker K Ji YL Hong XM Wang XB. Antenatal and neonatal factors contributing to extra uterine growth failure (EUGR) among preterm infants in Boston birth cohort (BBC). J Perinatol. (2021) 41:1025–32. doi: 10.1038/s41372-021-00948-4, 33589730 PMC7883994

[7] Hellström A Sigurdsson J Löfqvist C Hellgren G Kistner A. The IGF system and longitudinal growth in preterm infants in relation to gestational age, birth weight and gender. Growth Hormon IGF Res. (2020) 51:46–57. doi: 10.1016/j.ghir.2020.02.002, 32114373

[8] Li X Yu B Wu X Zhang J Jia C Wang Z . Associations between placental insulin-like growth factor-1 gene expression, DNA methylation and intrauterine growth restriction. Health. (2020) 12:270–80. doi: 10.4236/health.2020.123022

[9] Xiao WQ Zhang LF He JR Shen SY Funk AL Lu JH . Comparison of the INTERGROWTH-21st standard and a new reference for head circumference at birth among newborns in southern China. Pediatr Res. (2019) 86:529–36. doi: 10.1038/s41390-019-0446-0, 31158843

[10] Cebe FS Tola EN Koşar PA Oral B. DNA methylation profiles of genes associated with angiogenesis in the samples of placenta in pregnancies complicated by intrauterine growth restriction. J Matern-Fetal Neo M. (2021) 34:2854–62. doi: 10.1080/14767058.2019.1671344, 31581866

[11] Levine TA Grunau RE McAuliffe FM Alderdice FA. Early psychosocial development of small for gestational age and intrauterine growth-restricted children: a systematic review. J Perinatol. (2019) 39:1021–30. doi: 10.1038/s41372-019-0369-y, 30967654

[12] Hoon LH Kyu NO Sil CY. Neonatal outcomes of very low birth weight infants in Korean neonatal network from 2013 to 2016. J Korean Med Sci. (2019) 34:e40 30718992 10.3346/jkms.2019.34.e40 PMC6356024

[13] Vizzari G Morniroli D Tiraferri V Macchi M Gangi S Consales A . Postnatal growth of small for gestational age late preterm infants: determinants of catch-up growth. Pediatr Res. (2023) 94:365–70. doi: 10.1038/s41390-022-02402-3 36460739 PMC10356607

[14] Beghetti I Magno D Benvenuti E Aceti A Corvaglia LT. Risk factors for postnatal growth faltering and undernutrition at discharge in very preterm infants: a retrospective study applying the ESPGHAN consensus definitions. Nutrients. (2026) 18:286. doi: 10.3390/nu18020286, 41599899 PMC12844897

[15] Rochow N Landau-Crangle E So HY Pelc A Fusch G Dabritz J . Z-score differences based on cross-sectional growth charts do not reflect the growth rate of very low birth weight infants. PLoS One. (2019) 14:e0216048. doi: 10.1371/journal.pone.0216048, 31063464 PMC6504035

[16] Kang L Wang H He C Wang K Miao L Li Q . Postnatal growth in preterm infants during the first year of life: a population-based cohort study in China. PLoS One. (2019) 14:e0213762. doi: 10.1371/journal.pone.0213762 30973951 PMC6459511

[17] Vieira MC Relph S Persson M Seed PT Pasupathy D. Determination of birth-weight centile thresholds associated with adverse perinatal outcomes using population, customised, and intergrowth charts: a Swedish population-based cohort study. PLoS Med. (2019) 16:e1002902. doi: 10.1371/journal.pmed.1002902, 31539391 PMC6754137

[18] Chen YL Wang Y Chen ZK Xin QH Yu X Ma DF. The effects of rapid growth on body mass index and percent body fat: a meta-analysis. Clin Nutr. (2020) 39:3262–72. doi: 10.1016/j.clnu.2020.02.030, 32151438

[19] Toftlund LH Halken S Agertoft L Zachariassen G. Catch-up growth, rapid weight growth, and continuous growth from birth to 6 years of age in very-preterm-born children. Neonatology. (2018) 114:285–93. doi: 10.1038/s41598-023-50415-7 30011395

[20] Landau-Crangle E Rochow N Fenton TR Liu K Ali A So HY . Individualized postnatal growth trajectories for preterm pnfants. JPEN J Parenter Enteral Nutr. (2018) 42:1084–92. doi: 10.1002/jpen.1138 29419902

[21] Moreira PR Silveira MB Neves RO Nunes LM Bernarddi JR. Estimated energy and nutrient intake in complementary feeding methods in Brazilian infants: randomized clinical trial. Sci Rep. (2024) 14:13. doi: 10.1038/s41598-023-50415-7 38168148 PMC10761670

[22] Reyes SM Brockway M McDermid JM Chan D Granger M Refvik R . Human milk micronutrients and child growth and body composition in the first 2 years: a systematic review. Adv Nutr. (2024) 15:100082. doi: 10.1016/j.advnut.2023.06.005, 37315898 PMC10831887

[23] Amissah EA Brown J Harding JE. Protein supplementation of human milk for promoting growth in preterm infants. Cochrane Db Syst Rev. (2020) 2020:CD000433. doi: 10.1002/14651858.CD000433.pub3, 32964431 PMC8094919

[24] Baldassarre ME Panza R Cresi F Salvatori G Corvaglia L Aceti A . Complementary feeding in preterm infants: a position paper by Italian neonatal, Paediatric and Paediatric gastroenterology joint societies. Ital J Pediatr. (2022) 48:143. doi: 10.1186/s13052-022-01275-w, 35932061 PMC9354266

[25] Rochow N Fusch G Ali A Bhatia A So HY Iskander R . Individualized target fortification of breast milk with protein, carbohydrates, and fat for preterm infants: a double-blind randomized controlled trial. Clin Nutr. (2021). doi: 10.1002/14651858.CD000433.pub3 32446787

[26] Lindström L Ahlsson F Lundgren M, Bergman E Lampa E Wikström AK. Growth patterns during early childhood in children born small for gestational age and moderate preterm. Sci Rep (2019); 9:11578. doi: 10.1038/s41598-019-48055-x 31399623 PMC6688998

[27] A A Manohar N Hector D Bhole S Hayen A Eastwood J . Determinants for early introduction of complementary foods in Australian infants: findings from the HSHK birth cohort study. Nutrition J. (2020) 19:1–10. doi: 10.1186/s12937-020-0528-1, 32070350 PMC7029498

[28] de Beer M Vrijkotte TGM Fall CHD van Eijsden M Osmond C Gemke RJBJ. Associations of infant feeding and timing of linear growth and relative weight gain during early life with childhood body composition. Int J Obesity. (2015) 39:586–92. doi: 10.1038/ijo.2014.200 25435256

[29] Zhang L Lin J Liang S Sun J Gao N Wu Q . Comparison of postnatal growth charts of singleton preterm and term infants using World Health Organization standards at 40-160 weeks postmenstrual age: a Chinese single-center retrospective cohort study. Front Pediatr. (2021) 9:595882. doi: 10.3389/fped.2021.595882, 33791257 PMC8005644

[30] Wiley AS Joshi SM Lubree HG Bhat DS Memane NS Raut DA . IGF-I and IGFBP-3 concentrations at 2 years: associations with anthropometry and milk consumption in an Indian cohort. Eur J Clin Nutr. (2018) 72:564–71. doi: 10.1038/s41430-018-0108-z 29453428

[31] Kantake M Ikeda N Nakaoka H Ohkawa N Tanaka T Miyabayashi K . IGF1 gene is epigenetically activated in preterm infants with intrauterine growth restriction. Clin Epigenetics. (2020) 12:108. doi: 10.1186/s13148-020-00901-w, 32678007 PMC7364555

[32] Paulsen ME Marka N Lunos S Nagel EM Gonzalez Villamizar JD Nathan B . Insulin-like growth factor-1 and insulin-like growth factor binding protein-3 as early predictors of growth, body composition, and neurodevelopment in preterm infants. J Perinatol. (2024) 44:1617–23. doi: 10.1038/s41372-024-01933-3, 38561392 PMC11442679

[33] Paulsen ME Marka N Nagel EM Villamizar JDG Nathan BM Ramel SE. An exploratory study of clinical factors associated with IGF-1 and IGFBP-3 in preterm infants. Pediatr Res. (2024) 96:402–8. doi: 10.1038/s41390-023-02970-y, 38191823 PMC11228126

[34] Cao BY Peng YG Song WQ Peng XX Hu LX Liu ZL . Pediatric continuous reference intervals of serum insulin-like growth factor 1 levels in healthy Chinese children population-based on PRINCE study. Endocr Pract. (2022) 28:696–702. doi: 10.1016/j.eprac.2022.04.004, 35430364

[35] Pizzi C Cole TJ Richiardi L. dos-Santos-Silva I, Corvalan C, De Stavola B. Prenatal influences on size, velocity and tempo of infant growth: findings from three contemporary cohorts. PLoS One. (2014) 9:e90291. doi: 10.1371/journal.pone.0090291, 24587314 PMC3937389

[36] Vesel L Bellad RM Manji K. Feeding practices and growth patterns of moderately low birth weight infants in resource-limited settings: results from a multisite, longitudinal observational study. BMJ Open. (2023) 13:e067316. doi: 10.1136/bmjopen-2022-067316, 36792338 PMC9933750

[37] Zhang T Zhao L Ding W Ma J Zhang Y. The influence of perinatal and maternal factors on physical growth at 12 months in prematurely born infants treated in the neonatal intensive care unit: a retrospective chart review and a prospective cohort study. Int J Nurs Stud. (2020) 109:103656. doi: 10.1016/j.ijnurstu.2020.103656, 32593880

[38] Martin A Connelly A Bland RM Reilly JJ. Health impact of catch-up growth in low-birth weight infants: systematic review, evidence appraisal, and meta-analysis. Matern Child Nutr. (2017) 13:e12297–310. doi: 10.1111/mcn.12297, 27002681 PMC7158701

[39] Kim YM Lim HH Kim E Kim G Kim M So H . Exploring the genetic causes for postnatal growth failure in children born non-small for gestational age. J Clin Med. (2023) 12. doi: 10.3390/jcm12206508, 37892645 PMC10607479

[40] Fernandes AI Gollins LA Hagan JL Hair AB. Very preterm infants who receive transitional formulas as a complement to human milk can achieve catch-up growth. J Perinatol. (2019) 39:1492–7. doi: 10.1038/s41372-019-0499-2, 31570795

[41] Gao Y Luo JJ Zhang Y Pan CY Ren YJ Zhang J . Prenatal exposure to per- and polyfluoroalkyl substances and child growth trajectories in the first two years. Environ Health Perspect. (2022) 130:037006. doi: 10.1289/ehp9875, 35285689 PMC8919954

